# 여성 노인의 디지털 정보활용능력이 우울에 미치는 영향: 사회적 지지의 매개 효과를 중심으로

**DOI:** 10.4069/kjwhn.2023.08.30

**Published:** 2023-09-26

**Authors:** Ahyoung Lee, Soondool Chung

**Affiliations:** 1 Department of Social Welfare, Hallym University, Chuncheon, Korea; 1 한림대학교 사회복지학부; 2 Department of Social Welfare, Ewha Womans University, Seoul, Korea; 2 이화여자대학교 사회복지학과

**Keywords:** Depressive symptoms, Digital divide, Digital literacy, Older women, Social support, 우울, 디지털 격차, 디지털 정보활용능력, 여성 노인, 사회적 지지

## Abstract

**Purpose:**

The purpose of this study was to investigate the relationship between digital literacy and depressive symptoms, as well as the mediating role of social support in this relationship, among older women (60 years and older) in Korea.

**Methods:**

This study analyzed data from the User Experience Evaluation Survey, which was conducted by the Ewha Institute for Age Integration Research to improve the accessibility of digital information for older adults research from May to September 2020. Survey data on depressive symptoms, digital literacy, and social support were analyzed using descriptive statistics, Pearson correlation coefficients, and multiple regression.

**Results:**

The factors influencing depressive symptoms among older women included work status (B=–.19, *p*=.01), social support (B=–.17, *p*<.001), self–rated health (B=–.13, *p*=.003), and digital literacy (B=–.10, *p*=.005), which had an explanatory power of 33%. In addition, social support played a mediating role in the relationship between digital literacy and depressive symptoms (B=–.05, SE=.02; 95% CI, –.09 to –.02).

**Conclusion:**

The findings of this study support the need to develop and apply interventions that promote digital literacy among older women to mitigate depressive symptoms by increasing social support.

## Introduction

전 세계적으로 디지털 기술의 변화는 급속하게 이루어지고 있고, 그 예로 생성형 인공지능의 등장을 들 수 있으며, 이로 인하여 매우 빠른 속도로 교육, 여가, 의료, 문화 등 전반적인 생활양식의 변화가 일어나고 있다[1]. Social Network Services (SNS)를 사용한 가족 및 지인들과의 교류, 뉴스 등 각종 정보의 교환, 음악이나 사진 등의 취미생활뿐 아니라 은행 업무, 병원 예약, 장보기 등 이제는 언제 어디서나 디지털 기기 없는 삶을 생각하기 힘들다. 이러한 변화로 인하여 삶이 편리해진 면이 있지만, 급속한 변화에 따라가지 못하는 사람들은 사회적 소외를 경험하게 되는 부정적인 면도 존재한다[2]. 고령층은 이러한 디지털 격차(digital divide)를 보이는 대표적인 집단이다. 한국지능정보사회진흥원의 2021 디지털 정보격차 실태조사 보고서에 따르면 우리나라 만 55세 이상 고령층의 모바일 스마트 기기 보유율은 81.6%에 달하고 있지만, 고령층의 디지털 정보화 역량 수준은 2021년 비고령층의 53.9%에 머무르고 있으며 디지털 정보화 활용 수준도 72.3%에 머무르고 있다[3]. 고령층 중에서도 특히 여성은 디지털 소외를 겪는 대표적인 집단이라고 할 수 있다[2]. 위의 보고서에 따르면 고령층의 디지털 정보화 수준은 전 영역에서 여성보다 남성에서 높게 나타났으며[3], 선행연구마다 연령 기준이 조금씩 다르지만 55세 이상[4] 또는 60세 이상의 고령층을 대상으로 하는 연구에서[5] 고령층 여성의 디지털 이용경험 및 정보역량 수준이 고령층 남성보다 낮게 나타났다.

디지털 격차 문제는 단순히 디지털 이용능력 차이의 문제가 아닌 삶의 만족도와 우울 등 정신건강에 영향을 주는 문제이기에 더욱 중요하다[6,7]. 노년기에 접어들며 가족 관계의 축소, 은퇴 등으로 인한 사회적 관계 축소 등 노년기는 사회적 관계가 축소되는 시기여서 노인들은 소일거리 등으로 시간을 보내는 경우가 많아진다[8]. 이러한 상황에서 디지털 정보활용능력은 시간을 보낼 수 있는 유용한 도구가 될 수 있다. SNS나 소셜미디어를 통해 지인과 대화 나누기, 사진이나 영상 주고받기, 음악 공유하기, 동영상 시청 등을 할 수 있어 다양한 정보를 서로 주고받고, 취미나 여가생활을 공유할 뿐만 아니라 배움에 대한 욕구도 충족할 수 있다[4,8].

선행연구에서는 인터넷 사용이 사회적 관계망을 확장하고[9], 사회활동 만족도와 사회활동을 높이며[6], 디지털 정보활용 교육을 통하여 노인의 사회적 지지를 높일 수 있다고 하였다[10,11]. Lim 등[12]은 노인들을 대상으로 한 연구에서 디지털 정보활용능력이 개인의 사회 참여와 배제를 결정짓는 기준이 되며, 사회적 상호작용을 가능하게 하는 필수적인 역량이라고 하였다. 특히 여성일 경우 디지털 정보활용능력이 높을수록 사회활동 만족도가 높아지는 것으로 보고되었다[6]. 하지만 현재의 고령층이 살아온 시대에서 여성은 남성에 비해 사회 참여 기회가 적었으므로, 직장 내 업무를 위한 컴퓨터 사용 등의 디지털 교육을 받을 기회도 거의 없었다. 따라서 현재 여성 노인들은 디지털 활용능력을 통해 사회관계를 넓히는 데 제한적일 수 있으며, 향후 디지털 정보활용능력을 높임으로써 사회활동에 대한 만족도를 더욱 높일 수 있음을 시사한다[13].

한편 우울은 노년기에 흔히 발생하는 정신건강 질환으로, 2020년 노인실태조사에 따르면 한국 노인 중 약 13.5%가 우울을 경험하는 것으로 나타났다[14]. 이 조사에 따르면 노년층 중에서도 남성보다 여성이 우울증상을 갖고 있는 비율이 높았고, 연령이 높을수록 우울의 비율이 높은 것으로 나타났다. 많은 선행연구에서 사회적 지지가 노인의 우울을 감소한다고 하였다[15,16]. 특히, 가족과 배우자의 정서적 지지가 많을수록 우울이 감소하는 것으로 나타났다[15]. 이와 함께, 디지털 활용이 노인들의 우울을 낮춘다는 선행연구들도 있다[17,18]. Morris 등[19]은 이러한 디지털 활용이 노인들의 우울증을 감소시키는 사회적 연결의 매개 역할을 할 수 있다고 하였다. 하지만, Park과 Chung [20]은 디지털 정보활용능력이 우울 증상에 미치는 영향에 대한 최근 연구에서 사회적 네트워크의 매개 효과가 유의미하지 않음을 보고하기도 하였다.

**Figure 1.**
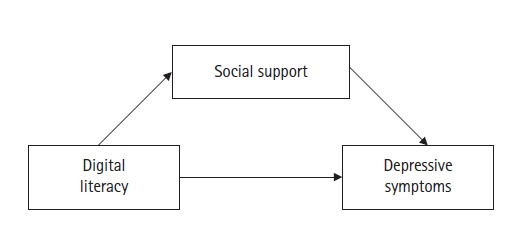
Conceptual framework of the study.

본 연구에서는 선행연구를 바탕으로 여성 노인의 우울에 대한 개입의 단초를 얻기 위하여 여성 노인의 디지털 정보활용능력이 우울에 미치는 영향을 알아보고, 이 관계에서 사회적 지지의 매개 효과를 알아보고자 한다. 선행연구를 기반으로 [21] Figure 1과 같은 연구모형을 제시하였으며, 구체적인 연구목적은 다음과 같다.

(1) 여성 노인의 디지털 정보활용능력이 우울에 미치는 영향을 파악한다.

(2) 여성 노인의 디지털 정보활용능력과 우울의 관계에서 사회적 지지의 매개 효과를 알아본다.

## Methods

Ethics statement: This study was a secondary analysis using anonymized data. The original study obtained informed consent from participants and adhered to the Declaration of Helsinki.

### 연구 설계

본 연구는 여성 노인의 디지털 정보활용능력이 우울에 미치는 영향을 알아보고, 이 관계에서 사회적 지지의 매개 효과를 검증하고자 이화여자대학교 연령통합고령사회연구소의 2020년 ‘노인의 디지털 정보접근성 향상을 위한 사용자 경험 평가조사’ 원시 자료를 이용한 이차 자료분석 연구로 매개 효과 분석 설계이다.

### 연구 자료

본 연구에서는 노인복지관 이용자들을 대상으로 노인의 디지털 이용에 대한 연구에서 수집한 자료를 이차적으로 사용하였다(unpublished literature) 원 자료는 유의적 표집방법을 이용하여 2020년 5월에서 9월까지 서울 시내 노인복지관 다섯 군데에서 수집하였다. 노인복지법 시행규칙 제24조에 따라 노인복지관 이용자는 60세 이상의 노인이므로, 본 연구는 60세 이상의 노인들을 대상으로 진행되었다. 복지관 게시판을 통하여 연구에 관심있는 참여자를 모집하였으며 본 연구의 목적을 이해하고 연구참여 동의서에 자발적으로 서명한 노인들만 연구에 참여하였다. 적절한 대상자 수는 G*Power 3.1.9.7 [22] 프로그램을 이용하여 산출하였다. 중간 효과크기 .15 [9,15], 검정력 .95, 유의수준 .05, 예측 변수 8개일 때 필요한 최소 대상자 수는 160명이었다. 예측 변수에는 디지털 정보활용능력과 사회적 지지 및 대상자의 특성 변수(연령, 결혼 여부, 교육 수준, 가구 소득, 주관적 건강 상태, 근로 상태)를 포함하였다. 데이터 수집은 노인복지관에서 연구 참여자가 직접 설문지에 응답하는 방식으로 이루어졌으며, 필요한 경우에는 연구보조원이 도움을 주었다. 총 376부의 설문지를 회수하였다. 본 연구에 필요한 자료는 익명 처리된 상태로 전달받아 분석을 시행하였으며 전체 자료에서 여성 노인 설문지 246부 중 누락된 응답이 포함된 설문지를 제외한 총 197부를 최종 자료 분석에 이용하였다.

### 연구 도구

#### 우울

우울은 단축형 Center for Epidemiological Studies Depression Scale Short Form (CES-D) 척도[23]의 한국판 도구[24]를 활용하여 측정하였다. 본 척도는 총 10문항으로 이루어져 있으며, ‘극히 드물었다(주 1일 미만; 1점)’에서 ‘대부분 그랬다(주 5일 이상; 4점)’의 4점 Likert 척도로 측정한다. 본 분석에는 합산 점수를 사용하였다(가능 점수 범위, 10–40점). 점수가 높을수록 우울 정도가 높은 것을 의미한다. 원 척도의 신뢰도는 .82였으며[22], 본 연구에서 Cronbach’s α는 .81이었다.

#### 디지털 정보활용능력

본 연구에서 디지털 정보활용능력은 컴퓨터, 인터넷, 모바일 등의 활용능력을 의미하며 Shin과 Lee [25]가 개발한 디지털 문해력 측정도구 중 ‘ICT 기본 역량’ 하위요인 5문항을 이용하여 측정하였다. 예시 문항으로 ‘나는 스마트 기기를 일상생활에 활용할 수 있다’, ‘나는 인터넷을 활용해 폭넓은 정보를 습득할 수 있다’, ‘나는 인터넷을 통해 다른 사람에게 정보를 제공할 수 있다’ 등이 있다. 각 문항들은 ‘전혀 그렇지 않다(1점)’에서 ‘매우 그렇다(5점)’까지 5점 척도로 구성되어 있으며, 분석에는 모든 문항의 평균 점수를 사용하였다(가능 점수 범위, 1–5). 점수가 높을수록 디지털 정보활용능력의 수준이 높은 것으로 해석한다. 원 도구의 신뢰도는 .86이었으며[25] 본 연구에서 Cronbach’s α는 .95였다.

#### 사회적 지지

사회적 지지는 Medical Outcome Study Social Support Scale (MOS-SSS) [26]를 Lim 등 [27]이 한국어로 수정 및 번역한 내용 중에서 정서적/정보적 지지 8문항을 사용하였으며, 여기에는 ‘사람들이 나를 보살펴주고 있다’, ‘사랑과 정서적인 지지를 받고 있다고 생각한다’, ‘개인사나 가정사에 대해 이야기할 사람이 있다’ 등이 포함되어 있다. ‘전혀 그렇지 않다(1점)’에서 ‘매우 그렇다(5점)’까지 5점 Likert 척도를 사용하였으며 모든 문항의 평균 점수를 분석에 사용하였다(가능 점수 범위, 1–5). 점수가 높을수록 사회적 지지 정도가 높음을 의미한다. Lim 등[27]의 연구에서 신뢰도는 .96이었으며, 본 연구에서는 .87이었다.

#### 사회인구학적 특성

본 연구에서는 주요 변수들 간의 관계를 보다 명확히 하기 위해 우울과 관련이 있는 사회인구학적 특성(연령, 교육 수준, 결혼 상태, 가구 월 평균 소득, 근로 상태, 주관적 건강 상태)을 통제변수로 사용하였다.

### 자료 분석 방법

자료는 IBM SPSS statistics ver. 27.0 (IBM Corp., Armonk, NY, USA)와 SPSS Process Macro를 이용하여 다음과 같이 분석하였다.

(1) 참여자의 인구사회학적 특성과 주요 변수의 특성을 빈도와 백분율, 평균과 표준편차, 왜도와 첨도로 분석하였다.

(2) 주요 변수 간의 상관관계를 Pearson correlation coefficients로 분석하였다.

(3) 디지털 정보활용능력과 우울의 관계에서 사회적 지지의 매개 효과를 검증하기 위해서 Hayes [28]가 제안한 SPSS PROCESS Macro Model 4를 사용하였다.

(4) 매개 효과의 유의도를 검증하기 위해서 부트스트랩 표본 5,000개를 추출하여 95% 신뢰구간(confidence interval, CI)을 분석하였다.

## Results

연구 참여자의 인구사회학적 특성을 살펴보면(Table 1), 평균 연령은 72.9세(표준편차, 4.84)였으며, 범위는 60세에서 88세였다. 교육수준은 고등학교 졸업 이하가 145명(73.2%), 대학교 졸업이 44명(22.2%), 대학원 졸업이 9명(4.5%)으로 나타났다. 현재 기혼 상태인 참여자는 104명(52.5%)이었다. 가구 월 평균 소득은 100만 원 미만이 51.5%로 가장 많았고, 100만–200만 원이 21.2%, 200만–300만 원이 15.2%로 그 뒤를 이었다. 근로 상태는 노인일자리사업 참여자가 54.0%로 가장 많았고, 무직/은퇴(30.3%), 무급 가족종사자(9.1%), 임금 근로자(4.5%), 자영업자(2.0%) 순이었다. 주관적 건강 상태는 보통이 53.0%로 가장 많았으며, 좋음(28.8%), 매우 좋음(8.6%), 나쁨(8.1%), 매우 나쁨(1.5%) 순이었다. 복지관에서 스마트폰이나 컴퓨터 활용수업을 들어본 적이 있는지에 대해서는 51.5%가 있다고 응답하였다.

### 주요 변수 기술통계

주요 변수의 기술통계는 Table 2와 같다. 연구 대상자들의 우울은 최소 10, 최대 34의 범위 안에서 평균 18.19 (표준편차, 5.29)로 약간 낮은 수준이었다. 디지털 정보활용능력은 최소 1, 최대 5의 범위에서 평균 3.24 (표준편차, 1.09)로 나타났고, 사회적 지지는 최소 1, 최대 5의 범위에서 평균 3.32 (표준편차, .87)로 두 변수 모두 중간 정도 수준인 것으로 나타났다. 변수의 정규성을 확인하기 위해 왜도와 첨도를 확인한 결과, 왜도가 ±2보다 작고 첨도가 ±7보다 작아 정규성 가정을 충족하였다고 판단하였다[29].

### 주요 변수 간의 관계

본 연구에서 대상자의 우울과 디지털 정보활용능력은 부적 상관관계(r=–.38, *p*<.001)가 있는 것으로 나타났고, 우울과 사회적 지지도 부적 상관관계(r=–.41, *p*<.001)를 나타냈다. 디지털 정보활용능력과 사회적 지지는 약한 양의 상관관계(r=.34, *p*<.001)가 있는 것으로 나타났다(Table 3).

### 디지털 정보활용능력이 여성 노인의 사회적 지지와 우울에 미치는 영향

먼저 회귀분석을 시행하기 전에 상승변량(variance inflation factor) 값을 확인하여 최종 분석에 투입된 모든 변수들의 다중공선성을 확인하였다. 그 결과 모든 변수의 상승변량 값은 1.14–1.26으로 다중공선성의 문제를 가지지 않는 것으로 확인하였다[30]. 구체적으로 회귀분석 결과를 살펴보면, 첫 번째 모델에서 사회적 지지 정도에 영향을 미치는 변수는 디지털 정보활용능력이 유일하였다(B=.29, *p*<.001). 즉, 디지털 정보활용능력이 높을수록 사회적 지지가 높아지는 것으로 나타났다. 사회적 지지를 설명하는 모델의 설명력은 15%였다(adjusted R2=.15).

여성 노인의 우울에 유의미한 영향을 미치는 요인은 주관적 건강상태(B=–.13, *p*<.01), 근로상태(B=–.19, *p*<.05), 디지털 정보활용능력(B=–.10, *p*<.01), 사회적 지지(B=–.17, *p*<.001)로 나타났다. 본 연구의 모델에 포함된 변수들이 우울을 설명하는 설명력은 33%였다(adjusted R2=.33) (Table 4).

### 디지털 정보활용능력이 우울에 미치는 영향에서 사회적 지지의 매개 효과

**Figure 2.**
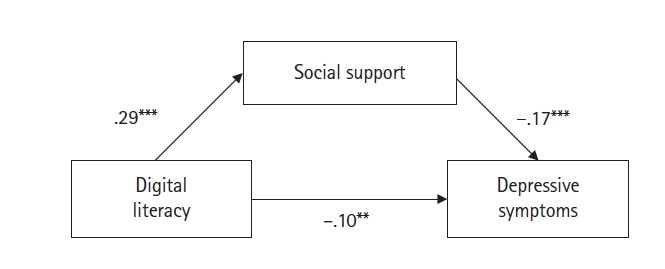
The mediation model of depressive symptoms. *p* <.05, **p <.01, ***p <.001.

부트스트래핑을 이용한 Process Macro의 매개 효과 검정 결과 디지털 정보활용능력이 우울에 미치는 영향에 대한 사회적 지지의 매개 효과(B=–.05, SE=.02; 95% CI, –.086 to –.022)는 95% CI에 0을 포함하지 않으므로 유의한 것으로 나타났다(Table 5). 따라서 디지털 정보활용능력이 높아지면 사회적 지지 정도가 높아지고, 우울이 감소하는 것으로 드러났다(Figure 2).

## Discussion

본 연구는 서울 지역 복지관 이용 여성 노인을 대상으로 디지털 정보활용능력이 우울에 미치는 영향과 그 관계에서 사회적 지지의 매개 효과를 검증하였다. 본 연구 결과 여성 노인의 디지털 정보활용능력이 우울을 낮춰주는 것으로 나타났으며, 이 관계에서 사회적 지지가 매개 효과를 갖는 것으로 나타났다. 이러한 결과는 디지털 교육 프로그램이 사회적 지지 수준을 높인다는 선행연구[9]와 유사하고 디지털 정보활용능력이 사회적 네트워크와 우울에 영향을 미친다는 선행연구[20]와 부분적으로 일치하는 결과이지만[20], 이에 더하여 여성 노인들을 대상으로 디지털 정보활용능력이 우울에 미치는 영향에서 사회적 지지의 매개 효과를 보여주었다는 점에서 선행연구와 차별된다. 디지털 정보활용능력으로 가족이나 친구에게 전화를 하고 메시지를 주고받으며 기존의 관계를 강화할 뿐만 아니라 새로운 관계를 형성할 수도 있어서, 이러한 사회적 관계가 우울의 감소에 도움을 준 것으로 해석된다.

본 연구 결과를 토대로 다음과 같이 논의하고자 한다. 본 연구의 주요 결과는 여성 노인의 디지털 정보활용능력과 우울의 사이에서 사회적 지지의 매개 효과를 검증한 것이다. 우울에 대한 적절한 개입방법으로 디지털 정보활용능력 교육을 들 수 있다. 많은 지자체, 도서관, 노인복지관에서 디지털 교육이 이루어지고 있지만 이러한 교육이 노인의 정신건강에 미치는 영향에 대한 효과성 평가는 거의 이루어지고 있지 않은 실정이다. Yoon 등[10]이 디지털 리터러시 교육이 노인의 우울을 감소하고 인지능력을 높이는 데 효과가 있으며, 실생활에서의 편리함 증진, 자신감 향상, 배움에 대한 욕구 향상, 사회적 관계망 강화 및 형성의 효과가 있다고 보고하였지만, 더 많은 효과성 평가 연구가 필요하다. 그러한 연구결과를 바탕으로 체계적인 디지털 교육 커리큘럼을 마련하고, 개인의 욕구에 맞는 수준별, 맞춤형 교육을 진행해야 할 것이다. 초고령 사회 진입을 목전에 둔 현 상황에서, 지자체, 복지관, 도서관, 경로당 등 지역사회 기반의 단체를 통한 노인 디지털 교육을 체계적으로 수행할 수 있는 인력도 개발해야 할 것이다.

또한, 본 연구에서 여성 노인의 디지털 정보활용능력과 우울의 관계에서 사회적 지지의 매개 효과가 검증되었다. 디지털 정보활용능력이 사회적 지지 수준에 영향을 미치는 것은 노인들이 인터넷 활용을 통해 사회적 관계망을 확장하고, 사회적 지지가 높아지며[11], 온라인을 통해 이미 알고 있던 친구나 지인과 소통함으로써 사회관계가 증가한다는 선행연구와 일치하는 것이다[10]. 즉, 여성 노인들이 스마트폰을 이용해서 자신의 가족, 친구, 지인들과 소통의 기회를 더 많이 가지고, 이에 더해 새로운 인적 네트워크를 가지게 되면서 사회적 지지가 높아지며, 그 결과 우울을 낮추는 것이다.

디지털 정보활용능력이 사회적 지지에 영향을 미치는 부분은 두 가지로 나누어 살펴볼 수 있다. 첫째, 여성들은 스마트폰을 이용해서 가족, 친구, 지인들과 소통하는 것을 즐겨하며, 디지털 정보활용능력이 높으면 지인과의 접촉 및 소통이 많아지고 이는 온라인 상에서뿐만 아니라 오프라인 만남 등으로도 이어져 대면과 비대면 모두를 통한 사회적 지지 수준을 높일 수 있다. 또 다른 가능성으로는, 온라인 상에서 새로운 관계를 구축하고 인간관계를 확장함으로써 사회적 지지 기반을 넓혀갈 가능성도 있다. 아직 우리나라 노인들이 온라인 상에서 모르는 사람들을 만나 새로운 관계를 맺는 행동에 대한 연구가 많지 않지만, Yoon 등[17]의 연구 결과를 참고해볼 만하다. Yoon 등[17]은 사회적 네트워크의 두 종류인 결속형(bonding)과 가교형(bridging) 사회자본의 개념을 이용하여 노인의 온라인 사회관계를 측정하였다. 결속형은 이미 알고 있는 사람들과의 사회적 네트워크로 가족, 친구 등 기존의 관계가 더욱 강화되는 사회자본이다. 반대로 가교형 사회자본은 서로 다른 배경을 가진 개인들이 자발적으로 관계를 만들 때 발생한다. Yoon 등[17]은 결속형 온라인 사회관계가 우울을 낮추는 효과가 있는 반면 가교형 온라인 사회관계는 우울에 영향을 미치지 않았다고 보고하였다. 즉, 온라인 상의 상호작용을 통하여 이미 알고 있는 지인들과의 결속을 다졌을 때, 이것이 사회적 지지 정도를 높여주고 우울을 낮춰주는 결과로 이어졌다는 것이다. 이러한 점에서 본 연구 결과를 해석해볼 수 있겠다.

본 연구의 제한점은 다음과 같다. 먼저 전국적인 확률 표집이 아닌 서울시 노인복지관 이용자를 대상으로 표본을 수집하였기 때문에 해당 결과를 일반화하기에는 한계가 있다. 추후 연구에서는 전국적인 표본과 노인복지관 비이용자를 포함한 좀 더 대표성 있는 표본을 대상으로 연구를 진행해야 할 것이다. 또한, 사회적 지지가 높을수록 자녀나 손자녀들에게 디지털 기기 이용방법을 배우는 기회가 증가하는 등의 이유로 디지털 정보활용능력이 높을 수 있다. 디지털 정보활용능력과 사회적 지지의 관계에 대한 좀 더 면밀한 연구가 진행되어야 할 것이다. 이러한 한계점에도 불구하고 본 연구는 디지털 소외계층으로 꼽히는 여성 노인들을 대상으로 디지털 정보활용능력과 우울의 관계에서 사회적 지지의 매개 효과를 검증하고, 여성 노인들의 우울 예방 및 개선을 위한 개입의 단초를 제공했다는 의의가 있다.

## Figures and Tables

**Table 1.** Demographic characteristics (N=197)

Variable	Mean±SD or n (%)
Age (year)	72.90±4.84 (range, 60–88)
Level of education	
≤High school	145 (73.2)
College	44 (22.2)
≥Graduate school	9 (4.5)
Marital status	
Married	104 (52.5)
Unmarried	94 (47.5)
Monthly household income (KRW)	
<1 million	102 (51.5)
1 million–2 million	42 (21.2)
2 million–3 million	30 (15.2)
3 million–4 million	12 (6.1)
>4 million	12 (6.1)
Work status	
Employed	9 (4.5)
Self-employed	4 (2.0)
Family business (unpaid)	18 (9.1)
Job program for seniors	107 (54)
Unemployed	60 (30.3)
Self-rated health	
Very good	17 (8.6)
Good	57 (28.8)
Moderate	105 (53.0)
Bad	16 (8.1)
Very bad	3 (1.5)
Have taken ICT classes	
Yes	102 (51.5)
No	96 (48.5)

KRW: Korean won (1 million KRW=roughly 800 US dollars); ICT: information and communication technology.

**Table 2.** Characteristics of the main variables (N=197)

Variable	Mean±SD	Possible range	Data range	Skewness	Kurtosis
Depressive symptoms	18.19±5.29	10–40	10–34	0.72	0.47
Digital literacy	3.24±1.09	1–5	1–5	–0.21	–0.75
Social support	3.32±0.87	1–5	1–5	–0.38	0.08

**Table 3.** Correlations among the key variables (N=197)

Variable	r (*p*)	
	Depressive symptoms	Digital literacy
Depressive symptoms	1	
Digital literacy	–.38 (<.001)	1
Social support	–.41 (<.001)	.34 (<.001)

**Table 4.** Multivariate regression model of depressive symptoms among older women (N=197)

Variable	Social support				Depressive symptoms			
	B	SE	t	*p*	B	SE	t	*p*
Age	.02	.01	1.54	.126	–.01	.01	–0.85	.394
Marital status^†^	.09	.13	0.68	.496	–.10	.07	–1.46	.146
Level of education	–.05	.06	–0.81	.419	–.02	.03	–0.68	.499
Monthly household income	.11	.05	1.97	.050	–.02	.03	–0.58	.562
Self-rated health	.05	.08	0.61	.542	–.13	.04	–3.04	.003
Work status^†^	.11	.14	0.78	.438	–.19	.07	–2.56	.011
Digital literacy	.29	.06	4.63	<.001	–.10	.04	–2.87	.005
Social support					–.17	.04	–4.42	<.001
F (*p*)	4.81 (<.001)				11.50 (<.001)			
Adjusted R^2^	.15				.33			

† The reference groups were marital status (married) and work status (working).

**Table 5.** Mediation analysis of the relationship between digital literacy and depressive symptoms (N=197)

Path	Direct effect	Indirect effect	Total effect	95% CI
Digital literacy → social support → depressive symptoms	–.10	–.05	–.15	–.086 to –.022

CI: Confidence interval.
